# Perching of Tengmalm's Owl (*Aegolius funereus*) Nestlings at the Nest Box Entrance: Effect of Time of the Day, Age, Wing Length and Body Weight

**DOI:** 10.1371/journal.pone.0097504

**Published:** 2014-05-14

**Authors:** Marek Kouba, Luděk Bartoš, Markéta Zárybnická

**Affiliations:** 1 Czech University of Life Sciences Prague, Faculty of Environmental Sciences, Department of Ecology, Suchdol, Czech Republic; 2 Czech University of Life Sciences Prague, Faculty of Agrobiology, Food and Natural Resources, Department of Animal Science and Ethology, Suchdol, Czech Republic; 3 Institute of Animal Science, Department of Ethology, Uhříněves, Czech Republic; University of Lausanne, Switzerland

## Abstract

The behaviour of the nestlings of nocturnal cavity-nesting species has relatively rarely been studied in detail because of problems connected with use of the technical devices required to provide long-term monitoring of individuals. However, long-term observation of nestling behaviour is crucial in order to identify different types of behaviour which may be caused by sibling competition at the end of nesting period. We studied behaviour of 43 Tengmalm's owl (*Aegolius funereus*) nestlings at 14 nests using a camera and a chip system. The nestlings perched at the nest box entrance from an average age of 28 days from hatching (range 24–34 days) until fledging, spending around 2 hours per day here in total, in periods ranging from a few seconds to 147 min (7.6±10.9 min, mean ± SD). We found that individual duration of perching at the nest box entrance was significantly influenced by nestlings' age and wing length and that the duration of perching at the nest box entrance significantly decreased with time of night. However, during daylight hours, time of day had no effect on either probability or duration of nestlings' perching. We suggest daylight perching at the nest box entrance results from nestlings' preparation for fledging, while individuals perching here during the night may gain an advantageous position for obtaining food from the parents; another possibility at all times of day is that nestlings can reaffirm their social dominance status by monopolizing the nest box entrance.

## Introduction

In bird species where parents deliver food to dependent young, there is frequently significant competition for resources (reviewed for example by [1]) and evidence is accumulating that parental provisioning behaviour can be influenced by nestlings' behaviour [2]–[6]. Altricial nestlings may improve their chances of being fed by seeking a particular position in the nest, reaching higher and closer to the visiting adult, and/or vocalizing first or with the greatest intensity (e.g., [5], [7]–[9]). Several studies on nestling behaviour show that advantageous position in the nest can improve a nestlings' chances, relative to those of its siblings, to obtain any food brought by the parents [7], [10]–[15]. Nestlings of cavity-nesting birds can improve their chances of obtaining food ahead of their siblings by positioning themselves as close as possible to the cavity entrance [16]–[18].

Such studies in owl nestlings are, however, scarce. Hofstetter & Ritchison [5] found out in Eastern Screech-owls (*Asio otus*) that the nestlings fed first by adults were those which started to beg significantly earlier, extended their beaks higher and closer to the adult, and called at higher rates and with greater volume than did their siblings. This result is in accordance with findings on vigilance of Barn owl (*Tyto alba*) nestlings by Roulin [19]. The more vigilant nestling was defined as the one that reacted first (i.e., that made a body movement) after a parent landed on the perch. These more vigilant individuals were as a rule fed first [19]. Because the extent of nestlings' potential activity is limited by their physical capabilities [20], [21], it is probable that their behavioural patterns and competition strategies will change with age. Thus, during the late nesting period when the nestlings are able to climb the wall from the nest floor to the nest entrance they may be able to perch at the entrance and wait for the parent there. Studies on such behaviour, especially in nocturnal birds of prey, are however lacking.

Tengmalm's owl (*Aegolius funereus*) is strictly night-active bird of prey with one peak of activity during the night in northern latitudes and two peaks in temperate latitudes [22], [23]. Differences in activity patterns between Finnish and Czech owls result from longer nights in central Europe compared to those in north Europe during breeding season [24]. This species naturally nests in natural tree cavities but also readily accepts nest boxes (e.g., [25]–[28]). It feeds mainly on small mammals [23], [29]–[31], and male provides nearly all food to the female from egg laying until she terminates her stay on the nest, and also, thereafter, for the young until independence (i.e., 5–9 weeks after leaving the nest; [32]–[35]). The male delivers prey at the nest entrance and in nearly all nest visits (99% of all cases) arrives with a prey. He either throws the prey into the nest cavity/nest box or gives it to the female or any nestling perching at the nest entrance, entering the nest cavity only exceptionally, most often when the female is not present [36]. The female incubates the eggs, broods the young, and remains almost continually in the nest cavity until the young are about 3 weeks old [23], [37].

Nestlings hatch at intervals of 1–2 days (at roughly the same intervals as the eggs are laid; [23], [26], [38]), and thus they differ in size. At the age of 3–4 weeks, the young are capable of thermoregulation and tearing up prey items for themselves. Starting at the 20th day of age, they show the first signs of ability to climb, are able to climb all the way to the cavity entrance at the age of 26–30 days, and leave the nest at the age of 27–38 days [23], [27], [34], [39], [40]. Wing length of nestlings continues to grow with age from hatching until the period after fledging (owlets fledge with incomplete feather growth), while body weight increases only to 3–4 weeks after hatching, and thereafter is essentially stable or may even decline [23], [41]. During this time (late nestling and post-fledging dependency period) the offspring may vocalize either in the presence and/or absence of the parents to solicit food [38], [42] as in other owl species (e. g., [43]–[46]).

In this paper we examine perching of Tengmalm's owl nestlings at the nest entrance and explore possible explanations for this behaviour. Using cameras, we obtained continuous data (24 hrs. per a day) on perching of Finnish and Czech nestlings at the entrance of artificial nest boxes. Because adult Tengmalm's owls are active only during the night (and roost during daylight; [23], [24], [47]), we predicted (i) that nestlings will perch at the nest box entrance and also leave the nest box (at fledging) only during the night time. We further predicted (ii) that duration of perching at the entrance will increase with the age of nestlings reflecting their improving ability to climb. Since wing length increases with age, (iii) perching at the nest entrance should also increase with the length of wing (with longer wings, in addition, conferring a greater ability to reach the entrance). Perching by nestlings at the nest box entrance may be motivated by gaining first access to food brought in by the parent and thus we also predicted (iv) that the duration of perching at the nest box entrance will decrease with the time of night as offspring will be gradually satiated. Finally, we expected the duration of perching at the nest box entrance will depend on nestlings' body condition (body weight), and in particular, (v) it will decrease with increasing body weight because satiated individuals (or individuals in better body condition indicating by higher body weight) will not need to wait at the nest entrance to obtain prior access to additional prey.

## Materials and Methods

### Study area

The study was conducted over two breeding seasons, in Finland during the breeding season of 2005 (63° N, 23° E) and during 2006 in the Czech Republic (50° N, 13° E). The Finnish site was situated in the Kauhava region of western Finland (50–110 m a. s. l.), covered ca 1300 km^2^, and included 500 nest boxes [48], [49]; the Czech site was situated in the Ore Mountains (730–960 m a. s. l.), covered ca 70 km^2^, and included 120 nest boxes. Nest boxes were square in section and made of wood, with base 25×25 cm, height 40 cm and an entrance hole 8 cm in diameter.

### Field procedures

Nestling behaviour was followed from April to August at nine nests (12% of the whole nesting population) in the Kauhava region in 2005 and at five nests (21% of the whole nesting population) in the Ore Mts. in 2006. For monitoring of nestlings we used special nest boxes equipped with camera and chip system (see below). Three such nest boxes were deployed at each study area. We were thus able to monitor three nest boxes simultaneously at any one time and, after all individuals had fledged from monitored nest boxes we were able to transfer the equipment to enable us to monitor later broods, allowing us to increase sample sizes, as above. Nests suitable for monitoring were selected randomly. Only nests located close to roads and paths were excluded to avoid drawing public attention to the boxes fitted with the technical apparatus. Once locations for monitoring had been selected, we replaced the original nest box with one fitted with the camera and chip system. The box replacements were carried out during hatching period, and thus, these nests were then monitored continuously from hatching until the last nestling left the nest.

The equipment used to monitor the nestlings' perching at the cavity entrance consisted of a camera (DECAM), a chip reader device, a movement data-logger, a movement infrared detector (KS96), and infrared lightning (IR diodes, SFH 485–2 880 nm, [50]). All nestlings were marked by chip rings (BR chip ring, BENZING), attached on the foot. A chip aerial affixed by the nest box entrance detected chip rings in its vicinity, i.e., only chip rings of the individuals appearing at or near (up to approximately 5 cm) the entrance of the nest box (the distance between the nest box floor and the bottom of the nest box entrance hole being 25 cm). The chip reader recorded the time and length of detection (beginning and end of detection) and the chip code of the ring. A camera was also installed inside the nest box opposite to the entrance. It was triggered by the infrared detector sensitive to movements in the nest box entrance. During the night, the nest box entrance hole was illuminated by infrared diodes at the time of the cameras' making photos. The time of detection was recorded by the movement data-logger and one to three photos were taken for each event. Using this equipment, we were able to record time and duration of each nestlings' perching at the nest box entrance.

In total, 43 nestlings were monitored: 32 from 9 nests in Finland (3.6±1.6 individuals per nest, mean ± SD; range 1–6) and 11 from 5 nests in the Czech Republic (2.2±1.2 individuals per nest, range 1–4). All individuals were weighed and the length of wing was measured at approximately weekly intervals during the afternoon hours (between 2–6 p.m.) which at least partially reduced possible fluctuations of nestlings' body weight since they are usually fed during the night time [24]. For the statistical analyses wing length was extrapolated to the age of 30 days from hatching for each individual and body weight was taken from the last weighing of each individual (i.e., 30.4±2.5 days from hatching, 2.3±2.0 days before fledging, mean ± SD). The age of the nestlings was in most cases based on the recorded date of hatching. In cases where the exact date of hatching was not recorded, the ages of such nestlings were estimated according to the growth curves (for wing length and body weight) valid for each of the studied populations [23], [41].

Owls were ringed under the Ringing Centre of the National Museum in Prague permit No. 329 and 942, were trapped and handled under the Ministry of the Environment of the Czech Republic permit No. 35016/02-OOP/8751/02, as well as under the Finish Museum of National History (licence No. 524) and all efforts were made to minimize suffering.

### Statistical analyses

When collecting the field data, we found that the nestlings are active and perching at the nest box entrance during the daylight as well as at night. Since this was in contrast with our expectation, we analysed daylight and night data separately; dividing the periods as I) night-time (i.e., Finland: 22:00–4:00 and Czech R.: 21:00–5:00) and II) day-time (i.e., Finland: 4:01–21:59 and Czech R.: 5:01–20:59).

All data were analysed with the aid of SAS System version 9.3 (SAS Institute Inc.). The analysis was made in three steps. Firstly, to verify that there were no differences in behaviour (total, average and individual duration of nestlings' perching at the nest box entrance hole) between study areas we used a multivariate General Linear Mixed Model (GLMM, PROC MIXED) with log-transformed duration of perching at the nest box entrance as a dependent variable with area (Finland and Czech Republic) and part of the day (day-time and night-time) nested within the area as fixed effects. The significance of each fixed effect in the GLMM was assessed by the F-test. To account for the repeated measures on the same individuals, the analysis was performed with individual fledging as a random factor. Least-squares means (LSMEANs) were computed for each class and differences between classes were tested by t-test. We used a Tukey-Kramer adjustment for multiple comparisons.

Secondly, to check for possible multicollinearity we calculated correlations between the individual variables involved (listed in Table 1). Correlation was found between wing length and body weight (0.53, P<0.0001) and wing length and time from hatching (−0.44, P<0.0001). We subsequently made a judgment of the extent of collinearity by checking related statistics, such as tolerance value or variance inflation factor (VIF), eigenvalue, and condition number following the approach of Belsley et al. [51] and using TOL, VIF and COLLIN options of the MODEL statement in the SAS REG procedure. Although, we did not find a support for exclusion either wing length, body weight or time from hatching from our models according to above mentioned indices for judgment of the extent of collinearity, we omitted body weight in models calculated both for daylight hours and night-time perching (I, II, see below). In both cases body weight was significant only when involved with wing length but nonsignificant when involved without wing length. Thus, we left only wing length in both models whose significance was much higher and therefore had to omit testing prediction (v). Time from hatching (i.e., nestlings' age) was also left in both models (I, II) since it was statistically significant (and the inter-correlation coefficients with other variables involved did not seem to be prohibitively large for them to be included alongside in the same model).

**Table 1:** Fixed effects used in the GLMMs (I, II) for the individual duration of nestlings' perching at the nest box entrance during the night (I) and daylight (II).

Fixed effect	Finland	Czech Republic
Number of eggs	6–8	4–6
Number of hatchlings	5–7	3–6
Date of hatching	11 April –16 June	8 May –9 June
Hatching order	1–6	1–4
Number of fledglings	1–6	1–4
Date of fledging	12 May –18 July	8 June –15 July
Time of fledging	7:48–4:33 hh:mm	9:34–22:17 hh:mm
Fledging order	1–6	1–4
Duration of period within the nest box from hatching	30–36 days	31–35 days
Time from hatching (nestlings' age)	24–36 days	27–35 days
Time of night when nestlings' perching at the nest box entrance was recorded	22:01–3:59 hh:mm	21:03–4:59 hh:mm
Time of daylight when nestlings' perching at the nest box entrance was recorded	4:00–21:59 hh:mm	5:02–20:58 hh:mm
Wing length extrapolated to the age of 30 days from hatching	90–138 mm	100–133 mm
Body weight at fledging	68–162 g	96–147 g
Age at the first perching at the entrance	24–30 days	27–34 days
Number of days with perching at the entrance	4–10 days	2–7 days
Number of individual perching events at the entrance	54–161	18–136
Total duration of all perching events at the entrance	4.3–22.6 hrs	2.8–11.8 hrs

(Data ranges for both study areas are shown).

In the third step of our analysis we tested the associations between the individual duration of perching at the nest box entrance (i.e., duration of each individual visit to the nest box entrance) and other variables (fixed and random effects) using a GLMM in each case; we analysed separately perching durations recorded (I) during the night (0.02 to 125.97 min, log transformed) and (II) during daylight (0.05 to 146.98 min, log transformed). To account for the use of repeated measures on the same individuals from the same nest box, analyses were performed using mixed model analysis with individual fledgling nested within nesting box, year and study area as random effects. Fixed effects employed within the models are summarised in Table 1.

We constructed the GLMMs I and II by entering first those factors which we expected to have an effect on individual duration of perching at the nest box entrance (time of night or day, time from hatching and nestlings' wing length) and then checking the model with addition of the factors which might also affect the result. If not specifically explained, non-significant factors (P>0.05) were dropped from the model and will not be mentioned any further. Where appropriate we tested interaction terms. Associations between the dependent variable and fixed effects were estimated by fitting a random coefficient model using PROC MIXED as described by Tao et al. [52]. We calculated predicted values of the log-transformed dependent variable and plotted them against the fixed effect with predicted regression line.

## Results

**Figure 1:**
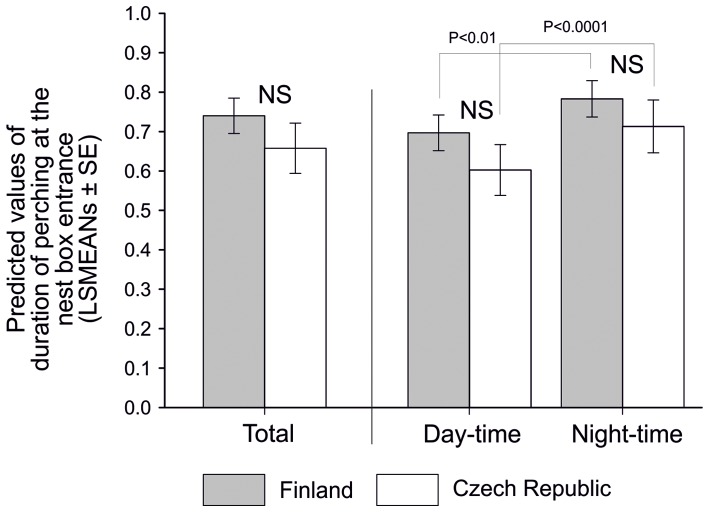
Comparison of log-transformed individual duration of nestlings' perching at the nest box entrance between and within the two study sites (Finland and Czech Republic) and parts of the day: Total (pooled day-time and night-time data), Day-time data, and Night-time data (LSMEANs ± SE).

The log-transformed duration of perching at the nest box entrance from Finland and the Czech Republic did not differ (GLMM, F_1, 79.2_ = 1.19, NS, Fig. 1). Part of the day nested within the area was significant (F_2, 3865_ = 17.84, P<0.0001) which reflected the difference between day-time and night-time both in Finland (t = −3.15, P = 0.009, Fig. 1) and the Czech Republic (t = −5.08, P<0.0001, Fig. 1). However, no significant difference was detected between the countries in periods defined as day (t = −1.26, NS, Fig. 1) or night (t = −0.83, NS, Fig. 1); we therefore subsequently pooled data from both areas for further analysis and did not further consider the effect of the area.

**Figure 2:**
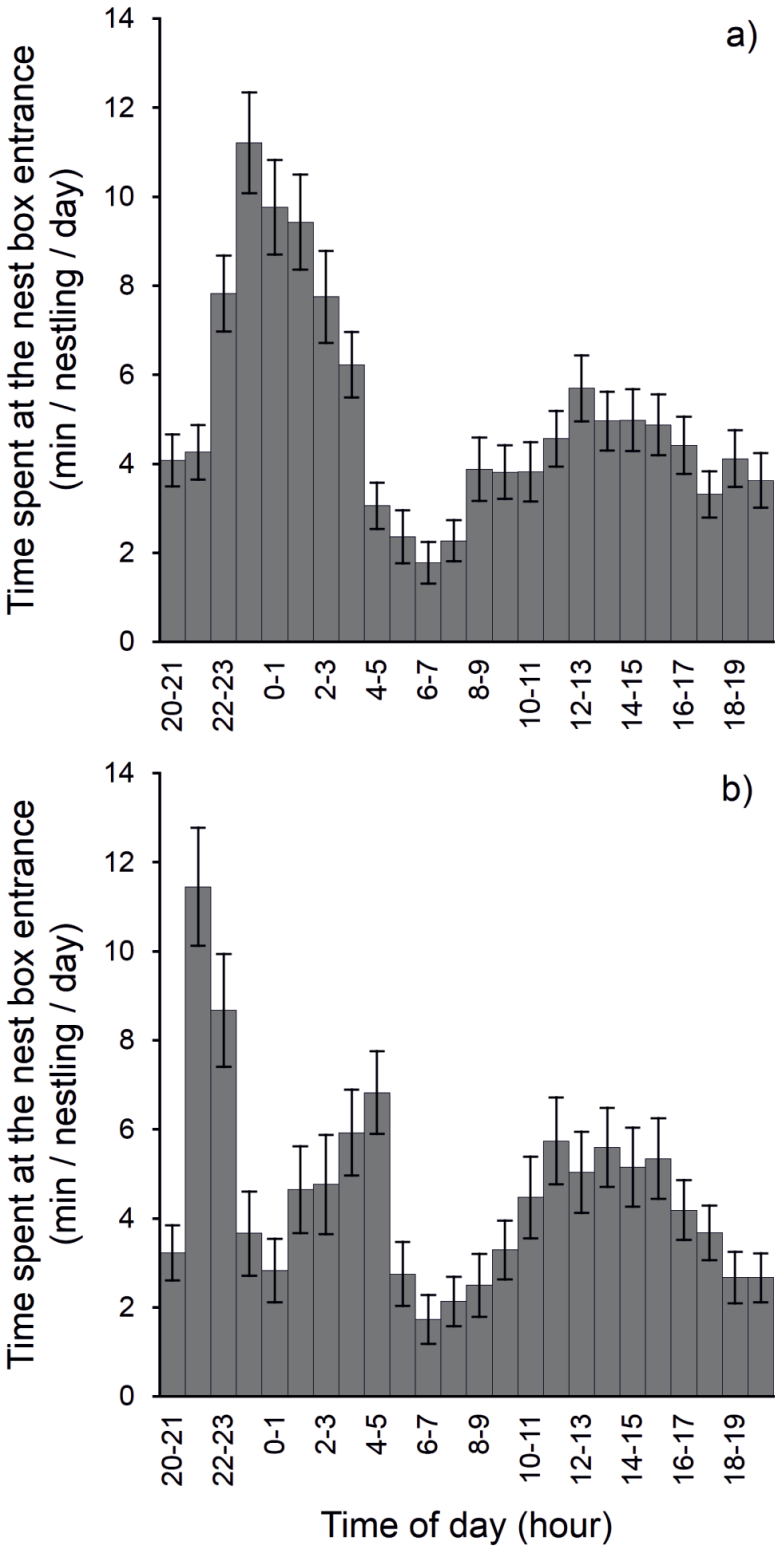
Perching duration of (a) Finnish, and (b) Czech nestlings at the nest box entrance throughout the day. Individual columns show the mean number (± SE) of minutes per nestling, day and nest.

In both study areas/seasons, nestlings began appearing at the nest box entrance at the age of 24–34 days after hatching (28.1±2.1 days, mean ± SD, n = 43). They usually appeared at the entrance in the order in which they had hatched and also in the same order in which they subsequently left the nest box, which happened on average 32.7±1.5 days after hatching (range 30–36 days). None of the nestlings left the nest box without spending some time at the cavity entrance. During the night-time they perched at the cavity entrance showing a single peak of activity in Finland (Fig. 2a) and two peaks of activity in the Czech Republic (Fig. 2b). However, nestlings also perched at the nest box entrance during daylight hours and with no conspicuous peak of activity in either of the study areas (Fig. 2a, b). Nestlings left the nest box (fledged) during both the night (n = 22) and the day (n = 21). No fledgling was recorded as returning to the nest after fledging.

The nestlings perched at the cavity entrance in varying intervals, ranging from 1 sec to 147 min (7.6±10.9 min, mean ± SD, n = 3868). In total, each nestling was observed perching at the entrance 32±16 times during nights and 58±25 times during daylights, i.e., 8.1±4.9 times per night and 12.4±7.6 times per daylight on average.

On average, each nestling spent 10.9 hours (±5.2 hrs, range 2.8–22.6, n = 43) at the entrance over a period of 5.6 days (±1.9 days, 2–10), an average which corresponds to a duration of 2.0±0.8 hours per day. This time was distributed fairly evenly across the 24 hour period: during the nights, the nestlings perched at the cavity entrance on average 1.0±0.5 hrs per night while during daylight, they perched at the entrance on average 1.3±0.6 hrs per day.

Mean time spent at the cavity entrance per perching event during the night (9.1±4.6 min, 1.2–20.9) was significantly longer than during the daylight (7.0±3.5 min, 1.4–17.3) (PROC UNIVARIATE, Wilcoxon signed-rank test: S = −295, p<0.0001, n = 43). The nestlings perching at the entrance often spread and folded their wings, and they always disappeared inside the nest box when the parents delivered prey.

### General perching patterns at the cavity entrance

**Figure 3:**
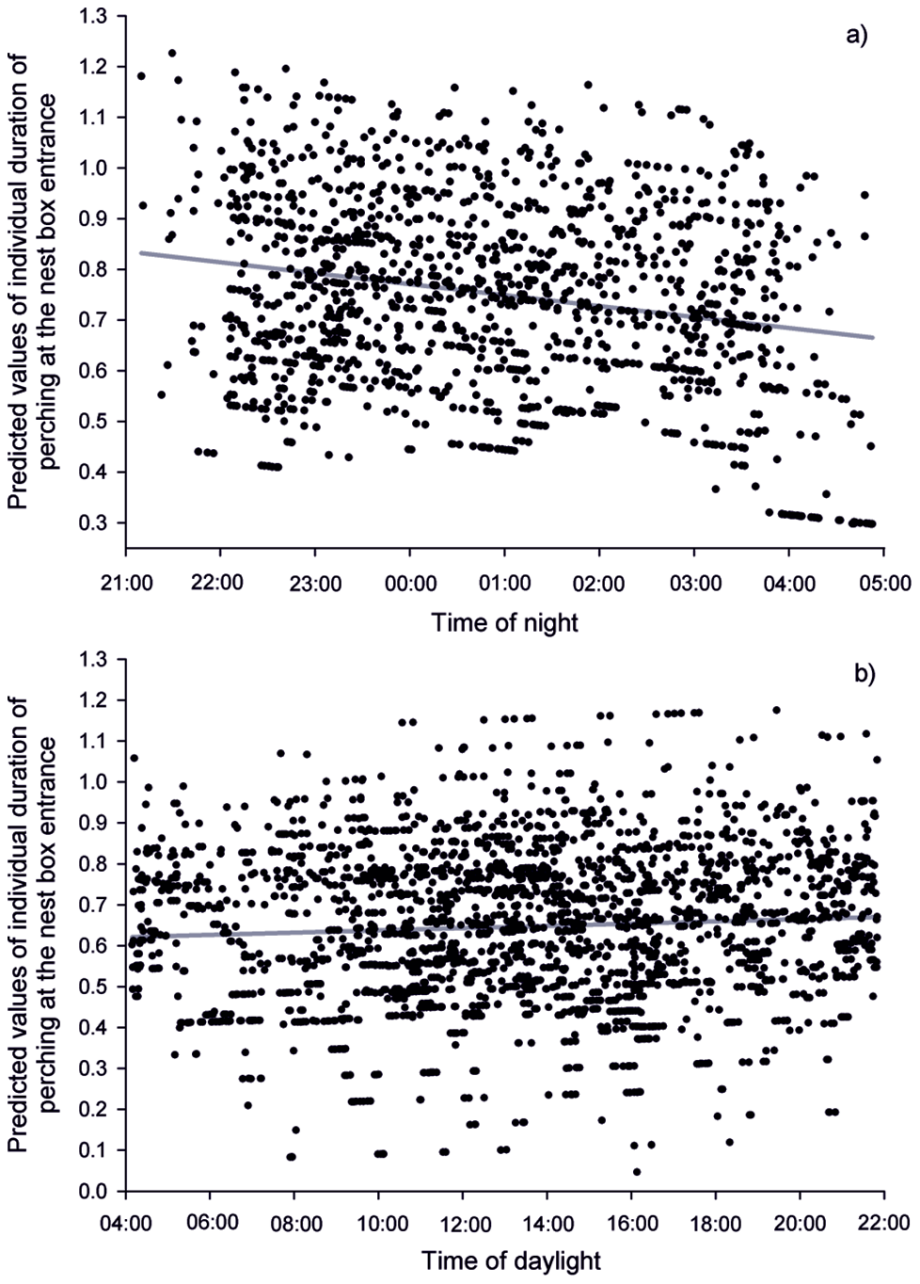
Predicted values of the log-transformed individual duration of nestlings' perching at the nest box entrance during a) night, and b) daylight plotted against the time of night and day, respectively.

**Figure 4:**
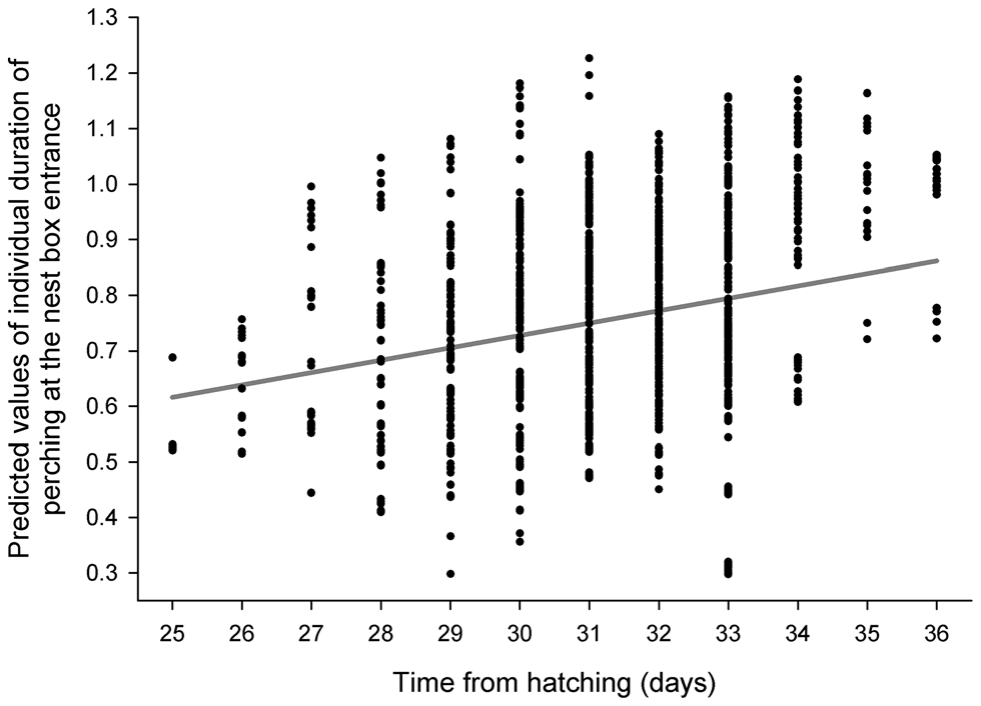
Predicted values of the log-transformed individual duration of nestlings' perching at the nest box entrance during the night plotted against the time from hatching (nestlings' age).

**Figure 5:**
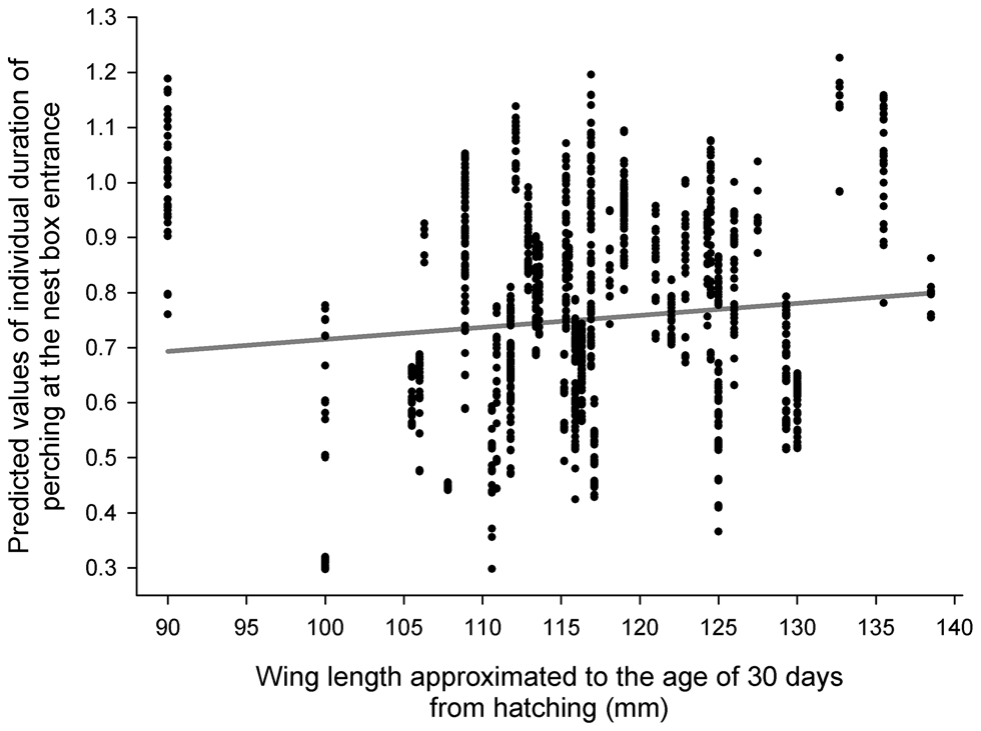
Predicted values of the log-transformed individual duration of nestlings' perching at the nest box entrance during the night plotted against the wing length extrapolated to the age of 30 days from hatching.

The results of the GLMM I for the individual duration of nestlings' perching at the nest box entrance during the night revealed that it was dependent on time of night (F_1, 1377_ = 10.38, P = 0.0013), nestlings' age (F_1, 1255_ = 45.13, P<0.0001) and wing length (F_1, 48.3_ = 6.93, P = 0.0113). Length of time spent perching at the nest box entrance decreased with the time of night (Fig. 3a), increased with increasing age (Fig. 4) and wing length (Fig. 5).

The results of the GLMM II for the individual duration of nestlings' perching at the nest box entrance during daylight revealed no formally significant effect of time of day with no real trend (F_1, 2490_ = 3.43, P = 0.0640, Fig. 3b, shown for illustration); however, time spent perched at the entrance was dependent on nestlings' age (F_1, 2184_ = 162.72, P<0.0001) and wing length (F_1, 46.8_ = 11.41, P = 0.0015). Duration of perching increased with increasing age and wing length (very similar to Figs. 4 and 5 and therefore not shown).

## Discussion

Our results showed that, throughout the late nesting period (1–2 weeks before fledging) Tengmalm's owl nestlings spend a total of about 8% of their time overall at the nest box entrance. This behaviour was shown by both Finish and Czech owl nestlings, suggesting the both populations have developed the same life-history trait. Contrary to our first prediction (i), nestlings perched at the cavity entrance during both the night and daytime, and also left the nest box (fledged) during both night and daylight periods; perching at the nest box entrance was however more frequent and for longer durations during the night than during the day. This perching behaviour (whether by night or by day) has rarely been described in nestlings of other birds of prey and owls although it has been observed for example in Little owls (*Athene noctua*) [53].

### Nestlings' activity at the cavity entrance during the night

According to our prediction (iv) we found that the individual duration of nestlings' perching at the nest box entrance decreased with the time of night. In Tengmalm's owl there is usually no prey stored in the nest during the latest stage of nesting because nestlings usually consume all prey immediately upon delivery by the parents ([23], Kouba & Zárybnická unpublished data). Thus, in perching at the nest entrance, hungry nestling(s) can be anticipating the arrival of the parents delivering prey in order to gain an advantageous position for obtaining food once it is brought. We suggest that the individual duration of nestlings' perching at the cavity entrance decreased through the night with increasing satiation of the nestlings – a conclusion consistent with observations in Tengmalm's owl fledglings that the probability of vocalization (begging for food) also decreased with the time of night during post-fledging dependence period [42].

Although we could not directly test the relationship between the nestlings' perching at the cavity entrance and receiving a prey item from parents we found that the activity patterns of Finnish and Czech nestlings at the nest box entrance showed peaks in activity corresponding to peaks in provisioning patterns of Finnish [23], [24] and Czech [22], [24], [36] Tengmalm's owls, respectively. These results support the hypothesis that perching of nestlings at the cavity entrance during the night is probably connected with gaining a food advantage for the individual perched there.

### Nestlings' activity at the cavity entrance during the daylight

Observations that nestlings also perch at the nest box entrance during the daylight were unexpected, since Tengmalm's owl adults are active exclusively at night [23], [24], [54]. Although, daylight activity has also been described in Barn owl nestlings [55] whose parents are also active almost exclusively at night [38], in our study the time spent at the nest box entrance during the daylight was appreciable because individual nestlings perched here for about an hour each, every day. In contrast to the pattern of this behaviour at night the amount of time nestlings spent perched at the cavity entrance during the day did not change with time of day. Half of all nestlings also left the nest box (fledged) during daylight hours.

We observed that nestlings perching at the cavity entrance often spread and folded up their wings which could be interpreted as deliberate exercising of the wings for future flying. There is usually little space on the nest box floor as a consequence of the presence of other siblings and the nestlings could gauge their flying muscles and preparedness for flight while perching at the nest box entrance. We suggest that unlike perching during the night, perching during daylight may thus be a preparation for leaving the nest box rather than positioning themselves for prior access to delivered prey.

### Factors influencing nestlings' activity at the cavity entrance

We found the individual duration of nestlings' perching at the nest box entrance increased with both age of nestlings and their wing length (ii and iii). These results were found for both parts of day (night and daylight). Tengmalm's owl nestlings were not physically able to climb to the cavity entrance before 24th day after hatching. However, as they got older and their wing length was increasing it became progressively easier for them to reach the entrance hole. We suggest that nestlings' ability to climb to the nest box entrance is also dependent on body size and condition as indicated by wing length.

We omitted to test prediction (v) because we excluded body weight from statistical models. However, the fact that body weight was in all cases nonsignificant when involved without wing length (result was found for both parts of the day – night and daylight) supports suggestion that body weight did not appear appropriate variable for assessing the degree of food-satiation of young and their long-term body condition as it has been recently shown [42]. This could be possibly explained by the fact that body weight increases only to 3–4 weeks after hatching, and thereafter is essentially stable or may even decline [23], [41]. Thus, wing length was statistically more influential in comparison with body weight during both parts of the day, which suggests importance of the wing length for assessment of the nestling's age and/or body size.

We propose different explanations of these findings for the two periods. It seems probable that older nestlings were likely to be able to out-compete their younger nest mates at any stage of the day or night in getting to the nest box entrance due to larger size (indicated by longer wings) and better climbing ability. During the night this could offer advantage in monopolizing prey items brought by the parents. On the other hand, during the daylight periods, when parents were not bringing food to the nest box [24], the older and larger siblings may have perched longer and more often at the entrance in preparation for fledging earlier than their younger and smaller nest mates [23]. It is also possible that by monopolizing the nest box entrance, older nestlings can reaffirm their social dominance status (in the same way as is reported for vocalisation by Barn owl nestlings [56], [57]).

We conclude that the activity patterns of nestlings shown in the frequency and duration of perching at the nest box entrance differed from activity patterns of adults because nestlings were active during the period of daylight as well as at night, in contrast to the strictly nocturnal habit of their parents. We suggest perching of nestlings at the nest box entrance during the daylight as a result of preparation for fledging while individuals perching at the nest box entrance during the night may gain prior access to prey items brought by the parent, and thus, have direct food advantage.
